# Hydrogen Generation using non-polar coaxial InGaN/GaN Multiple Quantum Well Structure Formed on Hollow n-GaN Nanowires

**DOI:** 10.1038/srep31996

**Published:** 2016-08-24

**Authors:** Ji-Hyeon Park, Arjun Mandal, San Kang, Uddipta Chatterjee, Jin Soo Kim, Byung-Guon Park, Moon-Deock Kim, Kwang-Un Jeong, Cheul-Ro Lee

**Affiliations:** 1 Semiconductor Materials Process Laboratory, School of Advanced Materials Engineering, Engineering College, Research Center for Advanced Materials Development (RCAMD), Chonbuk National University, Baekje-daero 567, Jeonju 54896, Republic of Korea; 2 Department of physics, Chungnam National University, 220 Gung-dong, Yuseong-gu, Daejeon 34134, South Korea; 3 Department of Polymer Nano Science and Technology, Chonbuk National University, Baekje-daero 567, Jeonju 54896, Republic of Korea

## Abstract

This article demonstrates for the first time to the best of our knowledge, the merits of InGaN/GaN multiple quantum wells (MQWs) grown on hollow n-GaN nanowires (NWs) as a plausible alternative for stable photoelectrochemical water splitting and efficient hydrogen generation. These hollow nanowires are achieved by a growth method rather not by conventional etching process. Therefore this approach becomes simplistic yet most effective. We believe relatively low Ga flux during the selective area growth (SAG) aids the hollow nanowire to grow. To compare the optoelectronic properties, simultaneously solid nanowires are also studied. In this present communication, we exhibit that lower thermal conductivity of hollow n-GaN NWs affects the material quality of InGaN/GaN MQWs by limiting In diffusion. As a result of this improvement in material quality and structural properties, photocurrent and photosensitivity are enhanced compared to the structures grown on solid n-GaN NWs. An incident photon-to-current efficiency (IPCE) of around ~33.3% is recorded at 365 nm wavelength for hollow NWs. We believe that multiple reflections of incident light inside the hollow n-GaN NWs assists in producing a larger amount of electron hole pairs in the active region. As a result the rate of hydrogen generation is also increased.

Over the past two decades, III-V compound semiconductor materials have been used extensively in the field of optoelectronics. In particular, various GaN-based device structures with outstanding features have been developed and realized for their significant applications as light emitting diodes (LEDs), lasers, solar cells, and photodetectors^1^,^2^. Besides these applications, metal-nitride semiconductors have also emerged as a new generation of materials for the applications of photoelectrochemical (PEC) water splitting and hydrogen generation^3^,^4^,^5^. The effective capture of sunlight and subsequent conversion into chemical fuels such as hydrogen has attracted considerable attention^6^,^7^,^8^,^9^,^10^,^11^,^12^. Compared to solar electricity, the use of chemical bonds to store solar energy promises significantly reduced device fabrication cost, as well as the cost associated with energy storage^13^,^14^. In this regard, hydrogen production by photoelectrolysis of water with semiconductor materials offers a clean, environmentally friendly process^13^,^14^,^15^.

A semiconductor that is to be used as a water splitting photoelectrode must meet several requirements related to its band gap and electronic energies. It also needs to be sufficiently stable that it is not damaged by the water splitting environment^16^. Both GaN and InN are known for their chemical stability. The potential difference between the hydrogen-producing half-reaction and the oxygen-producing half-reaction is 1.229 V. Thus, the band edges of InGaN (nearly 1.23 eV) can overlap the reduction and oxidation processes by providing the thermodynamically necessary energy to spilt water^16^. Moreover, among III-nitride semiconductors, InGaN is the only material whose energy bandgap can be tuned across nearly the entire solar spectrum^17^. In this study, we had dealt with InGaN/GaN multiple quantum wells (MQWs) structures grown on n-GaN 1-D nanostructers for PEC water splitting and hydrogen generation purpose. Also, GaN nanowires (NWs) offer a wide direct band gap (3.4 eV), high thermal conductivity (1.3 Wcm^−1^K^−1^), and highly saturated electron velocity, making themselves an obvious choice for improved device applications^18^,^19^,^20^,^21^,^22^.

PEC water splitting and hydrogen generation results using GaN based structures had been reported by many research groups. K. Maeda *et al*. reported H_2_ generation using a solid solution containing GaN and ZnO^23^. A detailed review of GaN and ZnO solid solution applied in water splitting can be found in ref. 24. Whereas K. Fujii *et al*. in 2005 showed n-type GaN as the working electrode for H_2_ generation using photoelectrochemical reactions^3^. In the same year Fujii and co-workers compared photoelectrochemical properties of p-type GaN with n-type GaN^4^. Very recently AlOtaibi *et al*.^16^ and Kibria *et al*.^25^ demonstrated water splitting and hydrogen generation using InGaN/GaN core/shell NW-based heterostructures. These uniaxial heterostructures were grown using plasma-assisted molecular beam epitaxy (PAMBE) technique. Moreover, AlOtaibi *et al*.^16^ reported an incident photon-to-current efficiency (IPCE) of ~27%. But J. Kamimura *et al*. found IPCE around 40%^26^. Whereas Kibira *et al*. reported an IQE of 60% by tuning the surface Fermi level of p-type GaN^27^. Furthermore J. Benton *et al*.^28^ and N. H. Alvi *et al*.^29^ reported IPCE of 46% and 67% respectively. In their review paper S. Zhao *et al*. extensively studied the evolution of IPCE in various GaN devices^30^. In this present scenario, to offer a polarization free larger effective area using m-plane InGaN/GaN heterostructures for maximum light harvesting and efficient hydrogen generation, a new growth method was applied to demonstrate a simpler but sophisticated InGaN/GaN heterostructure. The structures presented in this report InGaN/GaN MQWs on n-GaN 1-D nanostructers. InGaN/GaN MQWs were grown both on solid and hollow n-GaN NWs. InGaN/GaN MQWs grown on hollow n-GaN NWs were unique structures and an IPCE as high as ~33.3% is being reported which is not the highest recorded number for nanostructures though it is the best reported IPEC value for a hollow NWs system without utilizing any external photocatalyst. n-GaN NWs, both solid and hollow, were grown on Si (111) substrates using PAMBE but coaxial InGaN/GaN MQWs were grown on n-GaN NWs using metal organic chemical vapor deposition (MOCVD) process.

## Methodology

### Growth of Solid and Hollow n-GaN NWs

**Figure 1:**
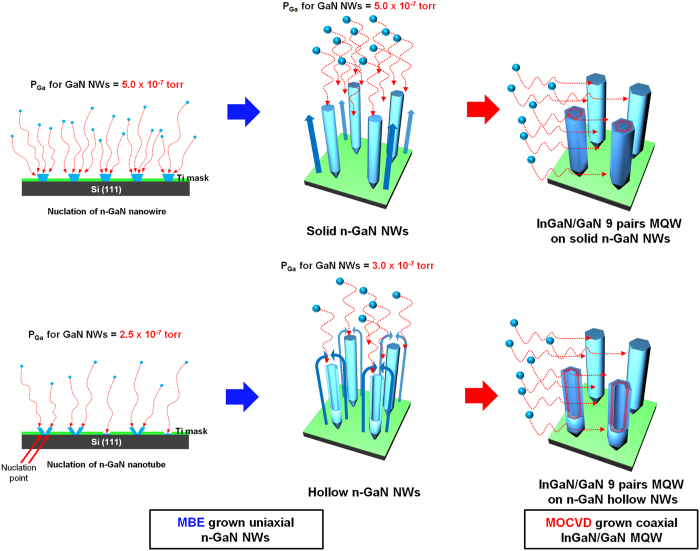
Schematic representation of the growth mechanism for both hollow and solid NWs. Schematic representation of the experimental procedures for growing coaxial InGaN/GaN MQWs on solid and hollow n-GaN NWs where a crosssectional representation of InGaN/GaN MQWs are shown for two front hollow NWs.

As mentioned earlier, a unique concept of hollow n-GaN NWs for efficient hydrogen generation was introduced in this study. Our approach was to make a comparative study between solid n-GaN NWs and hollow n-GaN NWs both grown on Si (111) substrates using PAMBE technique. To control the size and pitch of n-GaN NWs during growth, we used nano-patterned Si (111) substrate. To initiate, polystyrene (PS) beads of diameter 200 nm were first diluted in deionized water and then spin coated on Si (111) substrate. These monolayer-thick PS beads were then reduced to a diameter of 80 nm by reactive ion etching process, treated under oxygen plasma. Next, Ti films 15 nm thick were deposited over the PS beads using electron-beam deposition and finally, PS beads were removed by lift-off using ultrasonic bath. Patterned Si (111) substrates were then introduced into the ultra-high vacuum growth chamber of the PAMBE system equipped with a rf-plasma source for providing active nitrogen and standard Knudsen cells for solid source Ga and n-type dopant silicon. Prior to the growth of both solid and hollow n-GaN NWs, the temperature of the Si substrate was stabilized at 600 °C for ten minutes under nitrogen plasma atmosphere. Afterwards, Si-doped n-GaN NWs were grown on the patterned Si substrate at 750 °C. Growth recipes for both types of n-GaN NWs retained the same growth parameters except in the Ga flux. The Ga flux pressure was maintained at 5.0 × 10^−7^ torr and 2.5 × 10^−7^ torr for growing solid and hollow n-GaN NWs respectively. But to enclose the top part of the hollow nanowire, we increased the Ga flux to 3.0 × 10^−7^ torr for the last 10 minutes of growth process. Schematics representing the growth mechanism for solid and hollow n-GaN NWs are shown in Fig. 1. In case of the hollow NWs, the scarcity of Ga flux results GaN nucleation in periphery of the Ti masking pattern. After the initial edge nucleation interestingly the growth continues predominantly to be in the vertical direction instead of filling the patterned hole due to the higher activation energy of GaN than Ti. As also observed by F. Schuster *et al*. in their report, the less Ga presence in growth atmosphere should result into nanotubes by controlling the pattern density^31^. As shown in the Fig. 1, we increased Ga flux to close the top of the hollow NWs to avoid occurrence of stacking fault defect or any dislocation inside the nanohole while growing MQWs along the m-plane of the NWs. One can also observe some open hollow NWs or nanotubes in Fig. 1 because of limited Ga flux. On the other hand, affluent Ga flux results nucleation filling entire area of patterned hole which in turn reults into solid NWs.

Schematic representation of the experimental procedures for growing coaxial InGaN/GaN MQWs on solid and hollow n-GaN NWs where a crosssectional representation of InGaN/GaN MQWs are shown for two front hollow NWs.

### Growth of Coaxial MQWs on Hollow and Solid n-GaN NWs

Messanvi *et al*. in 2015 have commented on better light harvesting and higher internal quantum efficicency of MQWs over thick quantum well structure^32^. Moreover, MQWs having a narrow barrier layer (less than 4 nm) can demonstrate higher IQE due to carrier tunneling as reported by J. J. Wierer *et al*. in 2012^33^. So in the light of aforementioned findings, InGaN/GaN MQWs structures were designed further as active layers on PAMBE grown n-GaN NWs using MOCVD to find out how a through vacuum space inside a hollow n-GaN NW tunes the material and electrical properties of the devices. In the MOCVD system, nine pairs of InGaN/GaN MQWs were subsequently grown on n-GaN NWs using trimethylgallium (TMGa), trimethylindium (TMIn) and NH_3_ as the Ga, In, and N sources, respectively. Hydrogen gas was used as a carrier gas. The growth pressure was maintained at 400 Torr. We utilized the pulsed flow precursor method to grow the MQWs structures of InGaN wells and GaN barriers. Each InGaN well was grown at 640 °C for 200 seconds whereas GaN barrier was grown at 780 °C for 180 seconds. Finally, the n-GaN capping layer was grown for 10 minutes at 800 °C. Silane (SiH_4_) was used for n-type doping. During the growth, the flow rates of Ga, NH_3_, SiH_4_, and In were maintained at 0.3 sccm, 3 slm, 10 sccm, and 10 sccm, respectively. The thickness of GaN barrier layer was kept less than 4 nm whereas InGaN well was grown to be less than 3 nm for both the types of NWs. Schematic representations of the whole quantum structure for both the types of NWs are shown in Fig. 1.

### Optical Properties and PEC Measurements

Au/Ni metal electrodes were fabricated on both types of samples consisting of solid and hollow n-GaN NWs for photocurrent measurements. In order to record the PEC measurements, a three electrode system was used: a sample was used as the working electrode, Pt mesh as counter electrode, and Ag/AgCl (1 M KCl) as reference electrode. Incident light with a power density of 100 mW/cm^2^ was used as a light source during the water splitting experiment. PEC measurements were carried out in a homemade quartz chamber at room temperature. Before the measurement, deionized water was purged into the setup using Ar for 40 minutes. Using a thermal conductivity detector gas chromatography (TCD GC) system (Agilent-6890N), the amount of the evolved H_2_ was measured. Sampling was carried out using a vacuum syringe. High purity Ar (99.99999%) was used as a carrier gas.

### Characterization

A Hitachi S-7400 system was used for the FE-SEM study; it was operated at 15 kV and at a 13° tilt-view. A JEOL JEM-ARM-200F system operated at 200 KV was used in the HR-TEM study. Samples of the NW structures were prepared by coating with Platinum using a dual-beam focused ion beam (FIB, Quanta 3D FEG) technique with a beam current of 65 nA and a resolution of 7 nm at 30 KV. Single-crystal X-ray diffraction (XRD) measurements were performed using a Rigaku diffractometer equipped with a Cu-Kα radiation source. PL spectroscopy was carried out using a 325 nm line of a He-Cd laser as an excitation source at room temerature. CL measurements were taken at the applied accelerating voltage (V_a_) and beam current (I_b_) 10 KV and 1000 pA, respectively. For measuring photocurrent of the fabricated devices, we utilized a solar simulator (McScience Lab 100) as a light source. This light source generated white light with a maximum power density of 100 mW/cm^2^. A monochromator (Oriel Cornerstone 130) was used to provide the monochromatic light incident on the channel.

## Results and Discussion

### Structural Comparison between Solid and Hollow NWs

**Figure 2:**
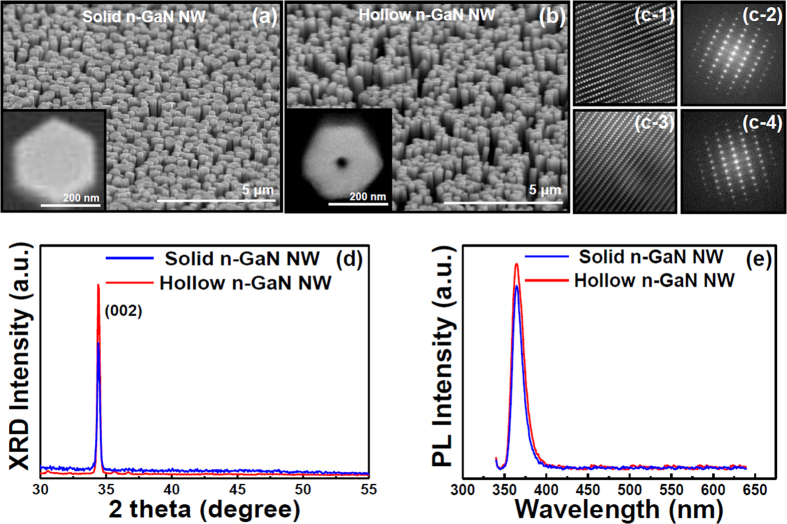
Tilt view FE-SEM images of (**a**) solid n-GaN NWs, (**b**) hollow n-GaN NWs; top view FE-SEM images of (a inset) solid n-GaN NW shows clear hexagonal shape and (b inset) a nano hole was noticed at the center of hexagonal hollow n-GaN NW; (**c-1,3**) and (**c-2,4**) show IFFT and SAED patterns of the solid and hollow n-GaN NW, respectively; (**d**) single-crystal XRD patterns of solid and hollow n-GaN NWs; (**e**) comparison of room temperature PL spectra of solid and hollow n-GaN NWs.

**Figure 3:**
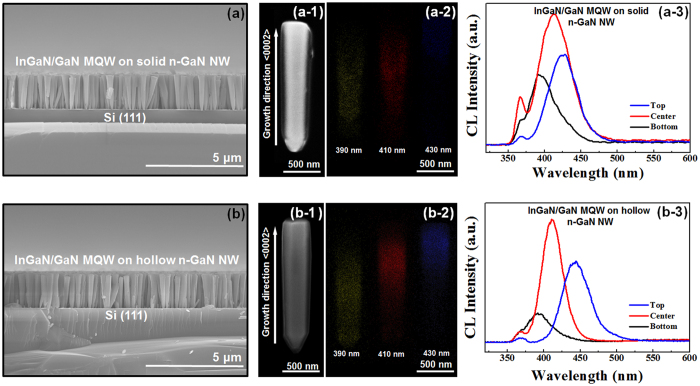
Cross sectional FE-SEM images of InGaN/GaN MQWs grown on (**a**) solid n-GaN NWs and (**b**) hollow n-GaN NWs; (**a-1**) and (**b-1**) show the FE-SEM images of single solid and hollow n-GaN NW, respectively, consisting of InGaN/GaN MQWs as active layers; (**a-2**) and (**b-2**) represent CL mappings of solid and hollow n-GaN NW, respectively at 390, 410 and 430 nm wavelength; (**a-3**) and (**b-3**) depict room temperature CL spectra of InGaN/GaN MQWs grown on solid and hollow n-GaN NWs, respectively.

Figure 2a,b show tilt view field emission scanning electron microscopy (FE-SEM) images of solid and hollow n-GaN NWs, respectively, after grown with PAMBE. As the Ga flux was made half during the growth of hollow NWs, their density also became less (Fig. 2b) compared to the solid n-GaN NWs (Fig. 2a). High resolution FE-SEM images of a single solid and hollow n-GaN NWs were shown in Fig. 2a,b, respectively. A nano sized hole (~less than 40 nm) at the center of the hexagonal hollow n-GaN NW was noticed (Fig. 2b inset). This inset image was taken after performing a FIB milling step which cut the n-GaN hollow NWs from the middle exposing the nanohole. The morphology of the n-GaN NWs was further revealed by high-resolution transmission electron microscopy (HR-TEM). The Inverse Fast Fourier Transform (IFFT) imaging (Fig. 2c-1,3) proved the fact that both types of nanowires were free of defects and dislocations and they were well-grown hexagonal structures as the representative selected-area electron diffraction (SAED) pattern shown in Fig. 2c-2,4 respectively for solid and hollow structures. Moreover, X-ray diffraction investigation was carried on the grown NWs to study the crystal quality. X-ray diffraction pattern showed a strong presence of the (002) plane for both solid and hollow n-GaN NWs confirming good crystallinity and material quality of both types of n-GaN NWs (Fig. 2d). The XRD results matched those of GaN listed in the Joint Committee on Powder Diffraction Standards (JCPDS) cards No. 50-0792. Therefore, this can be confirmed that there was no difference in the material quality of solid and hollow n-GaN NWs after they were grown with different rates of Ga flux using PAMBE. Room temperature photoluminescence (PL) measurements also proved the fact. There was almost no difference in PL emissions from solid and hollow n-GaN NWs (Fig. 2e). With these revelations, it would be fascinating to study further the effects of vacuum existent in hollow n-GaN NWs on the active layer’s material properties grown on NW body.

InGaN/GaN MQWs were grown on both solid and hollow n-GaN NWs using MOCVD and cross-sectional FE-SEM was further carried out. There was almost no difference noticed for both types of n-GaN NWs consisting of MQWs as active regions, from a morphological point of view (Fig. 3a,b). Figure 3a-1,b-1 show the FE-SEM images of a single solid and hollow n-GaN NW, respectively with InGaN/GaN MQWs grown on the NW body. Figure 3a-2,b-2 present their respective cathodoluminescence (CL) mapping. We believe that different CL mapping for InGaN/GaN MQWs was caused by difference in thermal conductivity between hollow and solid nanowire (Fig. 3a-2,b-2, respectively)^34^. In the aforementioned report, the investigators demonstrated a reduction in thermal conductivity by 33% by introducing a small hollow area of 1% of the total size of a nanowire. In lieu with their conclusion, we also expect a smaller thermal conductivy in case of grown hollow nanowires. Thus, lower In outdiffusion occurs as a result of lower thermal conductivy which in turn enhances InGaN/GaN MQWs crystal quality in hollow nanowires. In presence can be clearly noticed at the bottom, middle, and top part of both the NWs from CL mapping (Fig. 3a-2,b-2). But noticeably CL mapping intensity is more in case of hollow nanowire as shown in the images caused by the lower In outdiffusion. Furthermore, room temperature CL emissions of middle area show a broader full width at half-maximum (FWHM) of CL peak for InGaN/GaN MQWs grown on solid n-GaN NWs (Fig. 3a-3) compared to MQWs grown on hollow n-GaN NWs (Fig. 3b-3) which attributed to better material quality of InGaN/GaN MQWs when grown on hollow n-GaN NWs. More crystalline property studies were shown in TEM data. Visibly only the middle part of both the nanowires which is m-plane, reveals better crystalline properties compared to top and bottom part because this area is mostly free of defects and strain as defects and strain are concentrated at the edges or corners of c and r-plane of nanowires^35^,^36^. Interestingly the top area CL emission of hollow nanowire shows a redshift to higher wavelength which is again demonstrated in PL data. This red shift likewise endorsed the uniform presence of In for hollow nanowires due to lower outdiffusion^37^.

**Figure 4:**
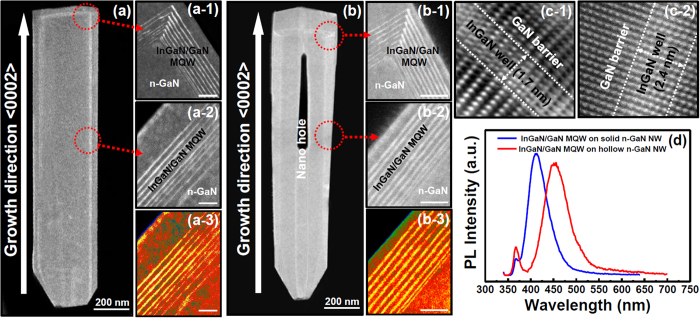
HR-TEM image of coaxially grown InGaN/GaN MQWs on (**a**) solid n-GaN NW and (**b**) hollow n-GaN NW; hollow n-GaN NW has a vacuum nano hole region inside; (**a-1~2**) and (**b-1~2**) show high magnification HR-TEM images of 9 pairs of InGaN/GaN MQWs grown on solid and hollow n-GaN NWs, respectively; (**a-3**) and (**b-3**) are the color representations of (**a-2**) and (**b-2**), respectively; (**c-1**) and (**c-2**) represent IFFT images of InGaN/GaN MQW grown on solid and hollow n-GaN NWs, respectively; (**d**) blue shift was noticed for the room temperature PL emission of InGaN/GaN MQWs grown on solid n-GaN NWs.

Figure 4a shows the HR-TEM image of a single solid n-GaN NW with InGaN/GaN MQWs grown on it as an active region, whereas Fig. 4b shows that of a single hollow n-GaN NW. Upon carefull inspection, one can notice the nano hole present throughout the length of nanowire in Fig. 4b. HR-TEM images give a closer look of the nine pairs of InGaN/GaN MQWs as Fig. 4a-1,2 and 3 show when grown on solid n-GaN NW and Fig. 4b-1,2 and 3 show when realized on hollow n-GaN NW. If the colored HR-TEM images are compared for InGaN/GaN MQWs grown on solid and hollow n-GaN NWs, one can observe that m-plane of the bottom layers of InGaN wells are not properly formed when grown on solid n-GaN NWs (Fig. 4a-3) whereas InGaN wells grown on hollow n-GaN NW exhibits nine pairs of well defined InGaN/GaN MQWs (Fig. 4b-3). We propose in this article that in the case of hollow n-GaN NWs, there is a vacuum nano hole created inside (Fig. 4b) and this nano hole plays an important role in determining the structural and material qualities of the InGaN/GaN MQWs grown as active layers on the hollow NW body. Due to this nano hole the thermal conductivity gets lowered in hollow nanowire which ultimately decreases the In outdiffusion during the growth of InGaN/GaN MQWs^34^. When GaN barriers are grown over InGaN wells at a comparatively higher temperature, there is a major possibility of In-Ga intermixing^34^ and, thus, deformation of InGaN wells. When the high resolution IFFT images were compared for InGaN/GaN MQWs grown on both solid and hollow n-GaN NWs, the phenomenon of In-Ga intermixing was clearly noticed for solid n-GaN NWs (Fig. 4c-1) and the width of the well was decreased to around 1.7 nm due to deformation. Figure 4c-2 showed the IFFT image of InGaN well and GaN barrier with almost no In-Ga intermixing and the width of InGaN well being around 2.4 nm when grown on hollow n-GaN NW because of the lower In outdiffusion. GaN barrier layers were measured to be 4.5 nm and 3.8 nm for solid and hollow NWs respectively. Lower In-Ga, intermixing also caused a red shift in the room temperature PL emission of InGaN/GaN MQWs when grown on hollow n-GaN NWs compared to solid NWs (Fig. 4d) which is consistant with abovementioned CL spectrum. HR-TEM results confirmed the fact that the nano hole inside the hollow n-GaN NW reduced the In outdiffusion by lowering the thermal conductivity of InGaN/GaN MQWs and thus improving their material and structural properties. Figure 4(a1, 2, 3,b1, 2, 3) were taken under 50 nm scale bar.

**Figure 5:**
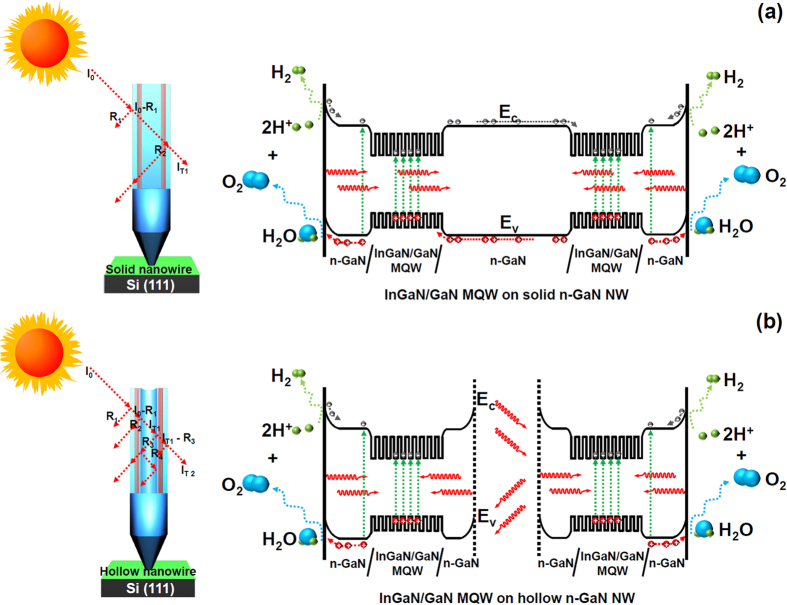
Schematic models to propose the reflection and absorption of incident light and electron hole pair generation inside the InGaN/GaN MQWs grown on (**a**) solid and (**b**) hollow n-GaN NWs. Band diagrams of both kinds of heterostructures are also presented to show how electron hole pair generation helps in hydrogen gas evolution in PEC water splitting experiment.

When light incidents on the InGaN/GaN MQWs grown on solid n-GaN NWs, the transition of carriers from the valence band to the conduction band happens throughout the heterostructure, from the surface n-GaN capping to the core n-GaN NW. The electron hole pairs are generated in every layer and the band diagram of the heterostructure containing solid n-GaN NW is shown schematically in Fig. 5a. Also, the fraction of the incident light absorbed within the heterostructure does not experience any further multiple reflections while traversing through the solid n-GaN NW (Fig. 5a). The excited carriers in the conduction band of solid n-GaN NW core travel a long distance in both ways before they are absorbed inside the InGaN wells on either side and then take part in radiative recombination (Fig. 5a). Thus, there remains a possibility that these carriers may get absorbed within the defects and non-radiative recombination centers of the solid n-GaN NW core itself before they reach the InGaN well. Whereas in case of InGaN/GaN MQWs grown on hollow n-GaN NWs, the transition of carriers from the valence band to the conduction band happens from the surface n-GaN capping to the nano hole on either side when light falls on the heterostructure (Fig. 5b). The fraction of incident light absorbed within the nano hole undergoes multiple reflections as shown in Fig. 5b. When the multiplied reflected light from the nano hole is absorbed by the coaxially grown InGaN wells on either side, the rate of electron hole pair generation is also multiplied. The models we have proposed in Fig. 5 explains why the InGaN/GaN MQWs grown on hollow n-GaN NWs can produce more photo-generated carriers compared to those grown on solid n-GaN NWs. How the generation of electron hole pairs is helping in the evolution of hydrogen during PEC water splitting experiment is also examplified in Fig. 5. This model is also reorted by Z. Lian *et al*. in 2015^38^.

### Optical Studies of Fabricated InGaN/GaN Heterostructures

**Figure 6:**
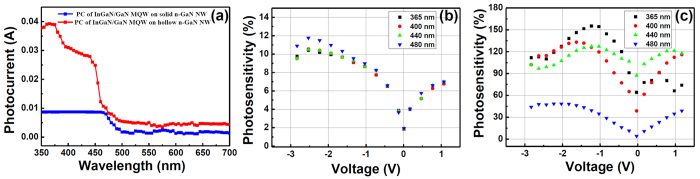
(**a**) Comparison of photocurrent recorded from InGaN/GaN MQWs grown on solid and hollow n-GaN NWs at an applied bias of −1.5 V; (**b**,**c**) show photosensitivity calculated at different wavelengths for InGaN/GaN MQWs grown on solid and hollow n-GaN NWs, respectively.

Figure 6a shows the comparison of photocurrents at a bias of −1.5 V, measured from InGaN/GaN MQWs grown on both solid and hollow n-GaN NWs. The photocurrent measured from the device consisting of solid n-GaN NWs represented a flat band nature of photocurrent value around 0.01 A within the wavelength of 350 to 475 nm. The photocurrent behavior of these coaxial InGaN/GaN MQWs are relatively better compared to the uniaxially grown InGaN/GaN core/shell NW based heterostructures^15^,^22^ as coaxially grown InGaN/GaN MQWs offer a larger active area. There was a fall in photocurrent value at around 500 nm wavelength which we believed was due to the limitation of the absorption range of the InGaN material we had grown (Fig. 6a). These deformed structures might take part in photocurrent generation. It was quite obvious that the improvement of the material quality of the InGaN/GaN MQWs grown on hollow n-GaN NWs, as concluded from the CL and HR-TEM results, helped in enhancing the photocurrent values compared to the wells grown on solid n-GaN NWs (Fig. 6a). Also, the participation of multiplied reflected light inside the nano hole enhanced photo carrier generation in case of MQWs grown on hollow n-GaN NWs (Fig. 5b). The photocurrent behavior also shows an increment around the wavelength 365 nm which can be attributed to hollow n-GaN core (Fig. 6a). Another increment can be seen around the wavelength 450 nm which we believe, is a result of photocurrent generated due the absorption by the InGaN well. Photocurrent results again confirmed the fact of the prominent existence of InGaN wells when grown on hollow n-GaN NWs.

**Figure 7:**
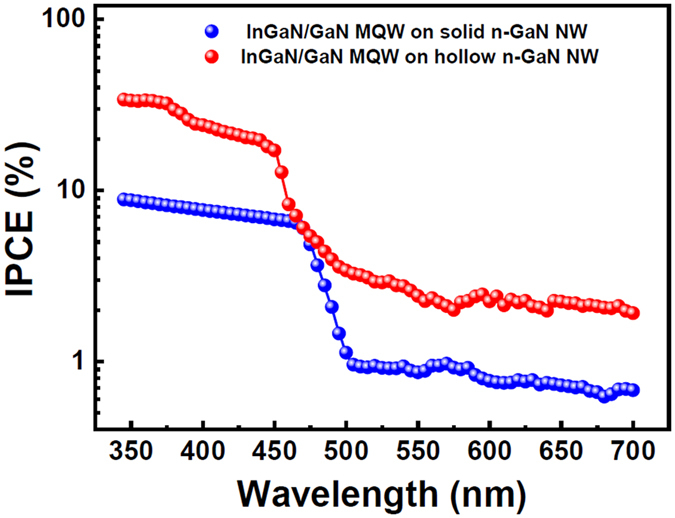
When IPCE data was compared for InGaN/GaN MQWs grown on both solid and hollow n-GaN NWs, a high IPCE value of ~33.3% was observed for the later.

When photosensitivity^39^ (I_P_/I_dark_) was recorded against applied bias at different wavelengths, photosensitivity values were found much higher for InGaN/GaN MQWs grown on hollow n-GaN NWs (Fig. 6c) compared to the wells grown on solid n-GaN NWs (Fig. 6b). Proper reasoning behind these phenomena goes with that of photocurrent already explained earlier in this article. Moreover, we measured the IPCE for these devices to further investigate the wavelength-dependent PEC properties of InGaN wells. The trend of IPCE was found likewise as in case of the photocurrent for InGaN/GaN MQWs grown on both solid and hollow n-GaN NWs (Fig. 7). IPCE measured for MQWs grown on hollow n-GaN NWs was about 33.3% at 365 nm wavelength. IPCE values also showed a sudden drop to 1.23% at 500 nm wavelength for MQWs grown on solid n-GaN NWs which was again due to the limitation of the absorption range of the InGaN material used. Photoresponses for both the type of devices gradually decrease after 500 nm wavelength. As also can be seen in Fig. 7, hollow n-GaN NWs show a higher IPCE value than the solid NWs. This IPEC investigation also confirms that the hollow NWs are better at photo harvesting compared to the solid NWs which is consistent with our previous results.

### PEC Studies on Fabricated InGaN/GaN Heterostructures

**Figure 8:**
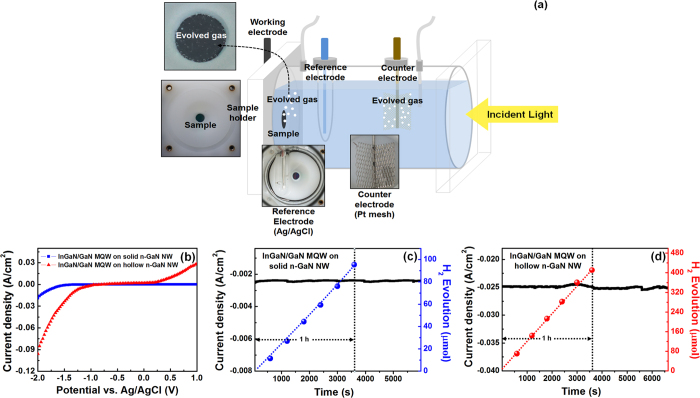
(**a**) Schematic of the PEC experimental setup, (**b**) Current density measured during PEC water splitting experiment from the InGaN/GaN MQWs grown on solid and hollow n-GaN NWs; at an applied bias of −1.0 V, (**c**,**d**) show hydrogen evolution rate and current density of the InGaN/GaN MQWs grown on solid and hollow n-GaN NWs, respectively. PEC water splitting and hydrogen generation experiment was carried out under illumination with a 300 W xenon arc lamp.

A schematic illustration of PEC experimental setup is shown in Fig. 8a. Figure 8b shows the measured current density as a function of applied potential for the InGaN/GaN MQWs grown on solid and hollow n-GaN NWs under illumination conditions with a xenon arc lamp (300 W). The bias was applied to the n-GaN working photoelectrode versus the Pt counter electrode, and measured from the Ag/AgCl reference electrode. The behavioral trend of the current is similar to that of photocurrents obtained from InGaN/GaN MQWs grown on both solid and hollow n-GaN NWs (Fig. 6a). In both cases, there was almost a four-fold increase in current for the wells grown on hollow n-GaN NWs compared to that grown on solid n-GaN NWs. Only an overall decrease in current density value was noticed due to the higher refractive index of water if compared with Fig. 6a. Around an applied bias of −1.5 V, a turn on and steep increase of current density were observed for the MQWs grown on hollow n-GaN NWs (Fig. 8b). This behavior of the biased channel can be correlated with the enhancement of the photosensitivity of the same structure at an applied bias of −1.2 V (Fig. 6c). During the PEC water splitting and hydrogen generation experiment, ideally there should not be any applied voltage as the band edges of InGaN wells can straddle the oxidation and reduction potentials of water. To get rid of external losses such as the resistive loss of the system, a bias of −1.5 V was applied in this experiment. A steady photocurrent was observed over a period of around two hours for both types of samples. Almost a 10 times increase of photocurrent was observed for the InGaN/GaN MQWs grown on hollow n-GaN NWs (Fig. 8d) compared to solid n-GaN NWs (Fig. 8c). The evolution of hydrogen gas from the Pt counter electrode over a period of one hour is also shown in Fig. 8c,d for respective sampling. How the hydrogen is being evolved photo-chemically is already represented in Fig. 5a,b. The gas generation rate is directly proportional to the photocurrent density. With better material properties and improved electrical responses, it was expected that the hydrogen generation rate would be higher for MQWs grown on hollow n-GaN NWs if compared with solid n-GaN NWs. After one hour, around 415 μmol hydrogen gas was generated by the former (Fig. 8d), whereas around 95 μmol hydrogen was generated by the latter (Fig. 8c).

## Conclusion

We report a new growth engineering where hollow n-GaN NWs were grown using PAMBE. Furthermore to offer a polarization free larger active area InGaN/GaN MQWs was grown coaxially on the n-GaN NWs using MOCVD. Moreover, excellent material properties were obtained for the MQWs grown on hollow n-GaN NWs. HR-TEM and CL studies proved the fact. Lower heat dissipation due to lower thermal conductivy in presence of nano hole inside the hollow n-GaN NW controlled the In outdiffusion of InGaN/GaN MQWs structures resulting better material and structural properties. Multiple reflections of incident light inside the nano hole influenced more and more photo excitation and multiplication of electron hole pairs, resulting an enhanced photocurrent and a higher rate of hydrogen evolution. To conclude, an IPCE as high as ~33.3% was recorded for the InGaN/GaN MQWs grown on hollow n-GaN NWs which is the highest reported value for any hollow NWs structures without any external photocatalyst.

## Additional Information

**How to cite this article**: Park, J.-H. *et al*. Hydrogen Generation using non-polar coaxial InGaN/GaN Multiple Quantum Well Structure Formed on Hollow n-GaN Nanowires. *Sci. Rep.* **6**, 31996; doi: 10.1038/srep31996 (2016).
